# Mucormicosis oral asociada a COVID-19 y diabetes mellitus: descripción de un caso

**DOI:** 10.7705/biomedica.6970

**Published:** 2024-03-31

**Authors:** Julio César Velasco, Ledmar Jovanny Vargas, Lorena García, Iván José Torres, Iván Camilo González

**Affiliations:** 1 Departamento de Investigación, Hospital Regional de la Orinoquía, Yopal, Colombia Hospital Regional de la Orinoquía Departamento de Investigación Hospital Regional de la Orinoquía Yopal Colombia; 2 Cirugía Maxilofacial, Hospital Regional de la Orinoquía, Yopal, Colombia Hospital Regional de la Orinoquía Hospital Regional de la Orinoquía Yopal Colombia; 3 Medicina de Urgencias, Hospital Regional de la Orinoquía, Yopal, Colombia Hospital Regional de la Orinoquía Hospital Regional de la Orinoquía Yopal Colombia

**Keywords:** mucormicosis, mucorales, tolerancia inmunológica, COVID-19, diabetes mellitus, mortalidad, mucormycosis, mucorals, immune tolerance, COVID-19, diabetes mellitus, mortality

## Abstract

La mucormicosis es una infección fúngica oportunista e invasiva, con una elevada tasa de mortalidad. Se ha detectado principalmente en pacientes con COVID-19, especialmente en personas con enfermedades concomitantes como la diabetes mellitus. La prevalencia de las mucormicosis es de 0,005 a 1,7 casos por millón de habitantes y ha ido en aumento en países como India y Pakistán; puede afectar diferentes órganos y su forma clínica refleja el mecanismo de transmisión. Entre las formas frecuentes están la rino-orbital-cerebral y la pulmonar, por ello, debe sospecharse mucormicosis en los pacientes con lesiones necróticas en mucosas o piel.

Se presenta el caso de un paciente con antecedentes de diabetes mellitus que fue diagnosticado con mucormicosis oral asociada a la COVID-19.

## Abstract (en)

Mucormycosis is an invasive opportunistic fungal infection with high mortality, mainly detected in people with COVID-19, especially those with underlying diseases such as diabetes mellitus. Mucormycosis prevalence is 0.005 to 1.7 cases per million inhabitants, and it has been increasing in countries like India and Pakistan. This mycosis can affect different organs, and clinical manifestations reflect the transmission mechanism. Frequent forms are rhino-orbital-cerebral and pulmonary. This disease should be suspected in patients with necrotic injuries on mucous membranes or skin.

We present a case of a patient with diabetes mellitus and diagnosed with oral mucormycosis associated with COVID-19.

La mucormicosis fue descrita por primera vez en 1976 por Ajello. Se describe como cualquier infección fúngica invasiva causada por especies del orden mucorales y entomoftorales ^1^*,* pueden afectar diferentes órganos y la forma clínica refleja su mecanismo de transmisión. Entre las formas más frecuentes están la rino-órbitocerebral (39 %), la pulmonar (24 %), la cutánea-subcutánea (19 %), la gastrointestinal (3 %) y la diseminada. Los síntomas más comunes incluyen invasión vascular, trombosis, isquemia tisular acompañada por infartos y necrosis tisular, por este último erróneamente se le ha llamado “hongo negro” ^2^^,^^3^.

La enfermedad por coronavirus (COVID-19) es causada por SARS- CoV2. La infección por COVID-19 en presencia de factores de riesgo como la diabetes mellitus, el uso de corticoides, la sinusitis no bacteriana, la infección por el virus de la inmunodeficiencia humana y los antecedentes de trasplantes favorecen la proliferación de infecciones fúngicas oportunistas como la mucormicosis ^4^.

Durante la pandemia se observó un aumento en el reporte de casos de mucormicosis asociadas a COVID-19 en países como India y Pakistán y en algunos latinoamericanos como Brasil, México y Argentina, con una prevalencia que ascendió a 14 casos por 100.000 habitantes y una tasa de mortalidad que oscila entre el 34 y el 70 % ^5^^-^^8^.

En el 2021, el Instituto Nacional de Salud reportó nueve casos de mucormicosis asociada a COVID-19 en Colombia. Los departamentos de Santander y Norte de Santander informaron dos casos cada uno y los de Antioquia, Bolívar, Córdoba, Valle del Cauca y Casanare, un caso por departamento. Las especies identificadas fueron: *Rhizopus oryzae* (40 %), *Rhizopus microsporus* (40 %) y *Mucor indicus* (20 %) ^7^.

El mecanismo fisiopatológico de la mucormicosis asociada a COVID-19 consiste en disminuir los potenciales de hidrógeno lo cual ocasiona la disociación de los complejos proteína-hierro. El hierro libre permanece en el plasma y sirve de insumo para el crecimiento del hongo. Otro mecanismo planteado es que la COVID-19 debilita la barrera defensiva del huésped, genera fagocitos disfuncionales y déficit de linfocitos ^8^.

El objetivo de este artículo es presentar el caso de un paciente con antecedentes de diabetes mellitus que fue diagnosticado con mucormicosis oral asociada a COVID-19.

## Caso clínico

Se trata de un hombre de 41 años con antecedentes de diabetes mellitus, que reporta baja adhesión al tratamiento farmacológico. Consultó por cuadro clínico de un día de evolución de disartria y ataxia. Además, manifestó cursar con una semana de fiebre no cuantificada, asociada a múltiples deposiciones diarreicas y disnea en reposo.

Los signos vitales estaban normales (presión arterial de 110/70 mm Hg, frecuencia cardiaca de 90 latidos por minuto y frecuencia respiratoria de 20 respiraciones por minuto). El paciente pesó 60 kg, midió 1,50 cm y su índice de masa corporal fue de 26,7 kg/m^2^.

**Figura 1:**
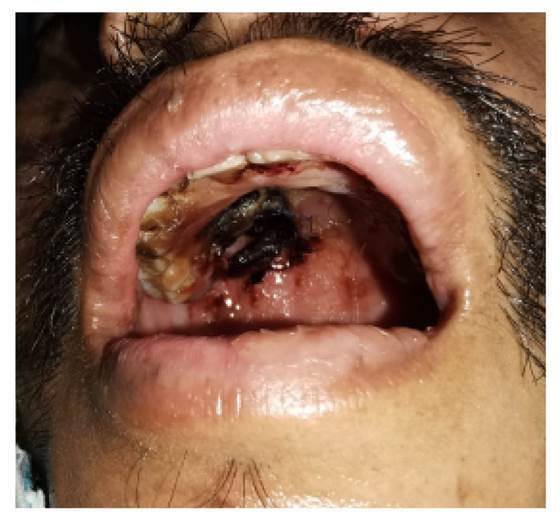
Área necrótica en paladar blando y duro con eritema perilesional

Durante el examen físico se observó una desviación de la comisura labial a la derecha y una lesión necrótica extensa en el paladar duro y blando (figura 1).

Entre los estudios realizados al ingreso, una tomografía computarizada simple (TC) de cráneo confirmó la presencia de un evento cerebrovascular isquémico agudo en la zona vertebro-basilar y arteria cerebral media derecha, además de pansinusopatía aguda. La glucosa en sangre fue de 614 mg/dl y la hemoglobina glicosilada de 7,9 %.

Se inició tratamiento con 4,5 g de piperacilina-tazobactam intravenosa cada seis horas, 18 UI de insulina glargina subcutánea al día, 8 UI de insulina cristalina subcutánea cada ocho horas y 40 mg de atorvastatina por vía oral por día.

Ante la evidencia de disnea se realizó una reacción en cadena de la polimerasa transcriptasa inversa (RT-PCR) para la amplificación del virus SARS-CoV-2 que resultó positiva, por lo que se prescribieron 50 mg diarios de prednisolona por vía oral, terapia física y respiratoria, sin necesidad de oxígeno complementario.

**Figura 2:**
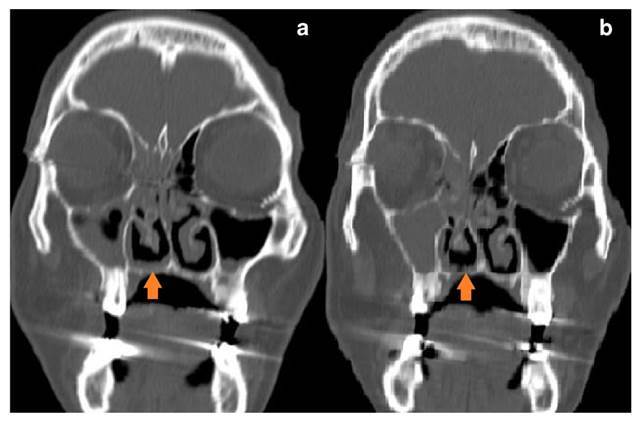
Tomografía computarizada: áreas de necrosis ósea focal y de partes blandas en la región parasagital derecha del paladar (aproximadamente de 4 mm) con tumefacción de las partes blandas adyacentes

**Figura 3:**
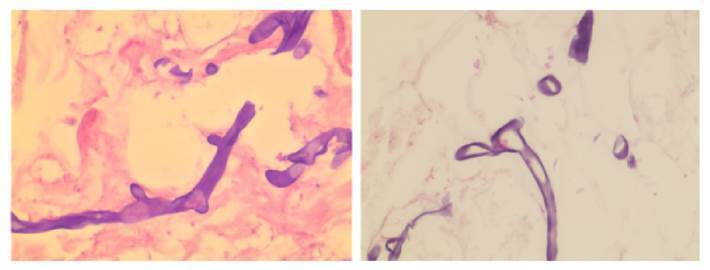
Hifas no tabicadas con ramificaciones (color fucsia) con tejido necrótico. Tinción de PAS (*Periodic acid-Schiff*), 100X.

En la TC del macizo facial se observó un defecto necrótico óseo focal y en parte del paladar blando (figura 2), mientras que en la de cuello se encontró una adenomegalia de 11 mm en la cadena yugulo-carotídea superior derecha. Se decidió practicar palatectomía parcial derecha con toma de biopsia (figura 3).

En el reporte histopatológico se informó la presencia de fragmentos de tejido necrótico con abundante cantidad de hifas no tabicadas, con ramificaciones en ángulo recto que invadían los vasos, sin fenómeno de Splendore-Hoeppli (cuerpos asteroides), compatible con mucormicosis. Se inició tratamiento con dosis de 300 mg de deoxicolato de anfotericina B en infusión por vía intravenosa durante seis horas por día, ya que era el único fármaco disponible al ingreso en la institución y se suspendió la antibioticoterapia.

Una vez lograda la estabilidad clínica y de la glucosa en sangre (125 mg/ dl), el paciente fue sometido a desbridamiento quirúrgico del paladar blando y duro; completó 42 días de tratamiento con deoxicolato de anfotericina B y al presentar evolución clínica favorable, con adecuada recuperación del estado neurológico, se le dio egreso hospitalario.

El paciente acudió al control ambulatorio y manifestó estar en buen estado general, con adecuada recuperación del déficit neurológico, sin lesiones isquémicas ni necróticas en el paladar.

### Consideraciones éticas

El paciente autorizó el uso de los datos clínicos y de las imágenes mediante un consentimiento informado.

## Discusión

Se presenta el caso de un paciente atendido en un centro hospitalario de Casanare por su importancia clínica y epidemiológica, con manifestaciones clínicas inespecíficas, antecedentes de diabetes mellitus no controlada e infección concomitante de COVID-19. Esto favoreció el desarrollo de un defecto necrótico óseo focal en el paladar duro y necrosis en el paladar blando, cuya etiología solo fue aclarada mediante el estudio histopatológico que permitió el diagnóstico definitivo de la mucormicosis.

Actualmente, el número de casos de mucormicosis a nivel mundial ha aumentado, lo que ha encendido las alarmas para diagnosticarlos oportunamente e identificar los factores de riesgo favorecedores de la enfermedad. Entre los factores de riesgo más conocidos están: la diabetes mellitus, la neutropenia y el tratamiento con corticoides ^6^^-^^9^, todos presentes en el caso clínico descrito, que junto con la COVID-19, se presume facilitaron la proliferación micótica.

Los estudios recientes reportan que los hombres están más predispuestos a sufrir de mucormicosis y el 76,3 % de los individuos infectados había recibido corticoesteroides como tratamiento para COVID-19 ^9^^-^^12^.

El caso en mención concuerda con lo encontrado en otras partes del mundo: sexo masculino, tener diabetes mellitus, haber recibido terapia con corticoesteroides y cursar con cuadro de COVID-19, lo que supone una disminución de los mecanismos de defensa para combatir este tipo infecciones. Según lo reportado por Prakash *et al*. ^11^, el sufrir de diabetes mellitus puede aumentar 7,5 veces las probabilidades de adquirir mucormicosis en comparación con otros factores de riesgo ^11^.

Aunque varios estudios aseguran que la diabetes mellitus no controlada es el principal factor de riesgo en la mucormicosis ^9^^-^^13^, la mortalidad estaba disminuyendo hasta antes de la pandemia de COVID-19. Rodríguez *et al*. ^13^ afirman que la letalidad depende de los factores predisponentes de cada individuo: 44 % en el sexo masculino, 45 % en diabéticos, 37,9 % en personas quemadas o con traumatismos penetrantes, 58,5 % en pacientes inmunocomprometidos y 55,6 % en aquellos con prescripción de corticoides. Este último, aunque ya había sido descrito como un factor de riesgo de la mucormicosis antes de la pandemia, probablemente influya en el aumento del número de casos en el mundo por su efecto inmunosupresor y por la elevación de la glucemia, al usarse como tratamiento en pacientes con COVID-19 -grave o crítico- como sucedió en el presente caso clínico.

Otros efectos bien conocidos causados por los corticosteroides son la disminución de la función de las células fagocíticas y la alteración de la capacidad migratoria, de ingestión y de fusión fagolisosómica de los macrófagos ^14^^-^^16^.

El manejo más asertivo para la mucormicosis se fundamenta en dos componentes: el tratamiento quirúrgico, en el cual es estrictamente necesario retirar el tejido necrótico existente para disminuir la probabilidad de sepsis fúngica y, la terapia antifúngica.

Respecto al tratamiento farmacológico, existen diferentes opciones y el medicamento de elección se basa en las capacidades locales o regionales. Según las recomendaciones de expertos el tratamiento debe brindarse en monoterapia. El fármaco de primera elección es la anfotericina B en dosis diarias de 0,5 mg/kg. Otros antifúngicos activos son los azoles, como el posaconazol, el isavuconazol y el voriconazol. El isavuconazol es menos hepatotóxico, pero ocasiona acortamiento del intervalo QTc; con la anfotericina B las dosis diarias no deben superar los 5 mg/kg debido a la toxicidad renal y, en tales casos, la dosis debe reducirse según sea necesario ^15^^,^^17^^-^^21^.

En el presente caso clínico, los médicos tratantes formularon el tratamiento con anfotericina B, ya que era el único fármaco disponible en la institución. Sin embargo, el medicamento fue tolerado adecuadamente por el paciente, sin que hubiera desarrollado ningún efecto secundario.

Con respecto al tratamiento quirúrgico, es vital considerar el desbridamiento agresivo de los tejidos necróticos afectados tan pronto como se sospeche el diagnóstico porque se reduce la probabilidad de desarrollar sepsis fúngica y se favorece la acción de los antifúngicos, pues estas áreas son inalcanzables solo con manejo farmacológico. Si hay dudas sobre el número de veces que se debe hacer el desbridamiento y en qué momento, lo aconsejable es realizar el número de intervenciones necesarias para remover cuanto antes el tejido necrótico donde ha proliferado el hongo ^18^^-^^21^.

## Conclusiones

Este reporte de caso es un ejemplo de mucormicosis asociada con COVID-19. Se encontró que la infección por SARS-CoV-2 es una condición clínica que exacerba la proliferación de infecciones oportunistas -como las micosis- en presencia de otros factores de riesgo que favorecen la enfermedad, principalmente la diabetes mellitus. Por lo tanto, se debe hacer lo posible para controlar medicamente las hiperglucemias de los pacientes con el fin de reducir la prevalencia y la mortalidad por mucormicosis.
